# Perfectionism, self-efficacy and mindfulness as predictors of test anxiety among university students

**DOI:** 10.1192/j.eurpsy.2024.885

**Published:** 2024-08-27

**Authors:** S. Tvrtkovic

**Affiliations:** Psychology, International University of Sarajevo, Sarajevo, Bosnia and Herzegovina

## Abstract

**Introduction:**

Test anxiety includes subjective experience of intense physiological, cognitive and/or behavioral symptoms during test-taking situations such as pacing, headaches, excessive feelings of fear, anger, troubles concentrating, sudden forgetfulness and negative self-talk. Especially students who are striving for flawlessness, have overly critical self-evaluations and beliefs that other expect perfection are sensitive to experiencing these feelings. On the contrary, individuals who believe in their ability, are present in the moment and are open to experiences tend to be more resilient to stressors and anxiety symptoms.

**Objectives:**

The aim of this study was to investigate perfectionism, self-efficacy, and mindfulness as predictors of test-anxiety among undergraduate and postgraduate students of different study fields.

**Methods:**

525 undergraduate and postgraduate students from the fields of Natural, Medical Sciences and Engineering, Social Sciences, Humanities and Art, and Economics, Business and Administration Studies participated in the study. A sociodemographic form, the Test Anxiety Inventory (TAI), 15-Item Five Facet Mindfulness Questionnaire (FFMQ-15), Scale of General Self-efficacy (GSES) and Frost Multidimensional Perfectionism Scale-Brief (FMPS-Brief ) were used. Descriptive statistics were used to show the sociodemographics of our sample, while correlational analyses were performed to asses the associations between the variables. To further validate the findings, multiple linear regression analyses were performed.

**Results:**

Higher perfectionistic evaluative concerns and lower self-efficacy showed associations with test-anxiety and were proven as predictors among undergraduate and postgraduate students. In addition, being younger and female in postgraduates, and having perfectionistic strivings and being Mindful-Observe in undergraduate students proved to be significant predictors of test-anxiety.

**Conclusions:**

The found associations of perfectionism and self-efficacy, as well as its predictor roles further validate the information we have in literature, but widen the sample to postgraduate students and various study fields which help us generalize the findings more. What adds the most value in theoretical and practical aspects is the findings associated with mindfulness. Mindfulness techniques are very popular intervention methods for anxiety related symptoms, and the positive relationship of Mindful-Observe with test anxiety opens new viewpoints to mindfulness-based interventions. Particularly focusing on Mindful-Observe while treating test-anxiety may yield better outcomes in alleviation of symptoms.

**Disclosure of Interest:**

None Declared

